# Ion selectivity and gating behavior of the CorA-type channel Bpss1228

**DOI:** 10.3389/fchem.2022.998075

**Published:** 2022-09-12

**Authors:** Yibo Zhu, Yu Wang, Yanjing Zhang, Mengjun Pu, Wenqian Miao, Mingran Bai, Rui Bao, Jia Geng

**Affiliations:** State Key Laboratory of Biotherapy, Department of Laboratory Medicine, Med-X Center for Manufacturing, West China Hospital, Sichuan University and Collaborative Innovation Center, Chengdu, China

**Keywords:** CorA channel, ion selectivity, magnesium ions, gating behavior, planar lipid bilayer

## Abstract

Magnesium is an essential element to sustain all forms of life. Total intracellular magnesium content is determined by the balance of magnesium influx and efflux. CorA is a divalent selective channel in the metal ion transport superfamily and is the major Mg^2+^ uptake pathway in prokaryotes and eukaryotic mitochondria. Previous studies have demonstrated that CorA showed distinct magnesium bound closed conformation and Mg^2+^-free states. In addition, CorA is regulated by cytoplasmic magnesium ions and its gating mechanism has been investigated by electron paramagnetic resonance technique and molecular dynamic simulations. Here, we report a study of the putative CorA-type channel Bpss1228 from *Burkholderia pseudomallei,* which has been shown to be significantly associated with pseudomallei infection. We expressed and purified the Bpss1228 in full-length. Subsequently, electrophysiological experiments further investigated the electrical characteristics of Bpss1228 and revealed that it was a strictly cation-selective channel. We also proved that Bpss1228 not only possessed magnesium-mediated regulatory property a remarkable ability to be modulated by magnesium ions. Finally, we observed the three-step gating behavior of Bpss1228 on planar lipid bilayer, and further proposed a synergistic gating mechanism by which CorA family channels control intracellular magnesium homeostasis.

## Introduction

**FIGURE 1:**
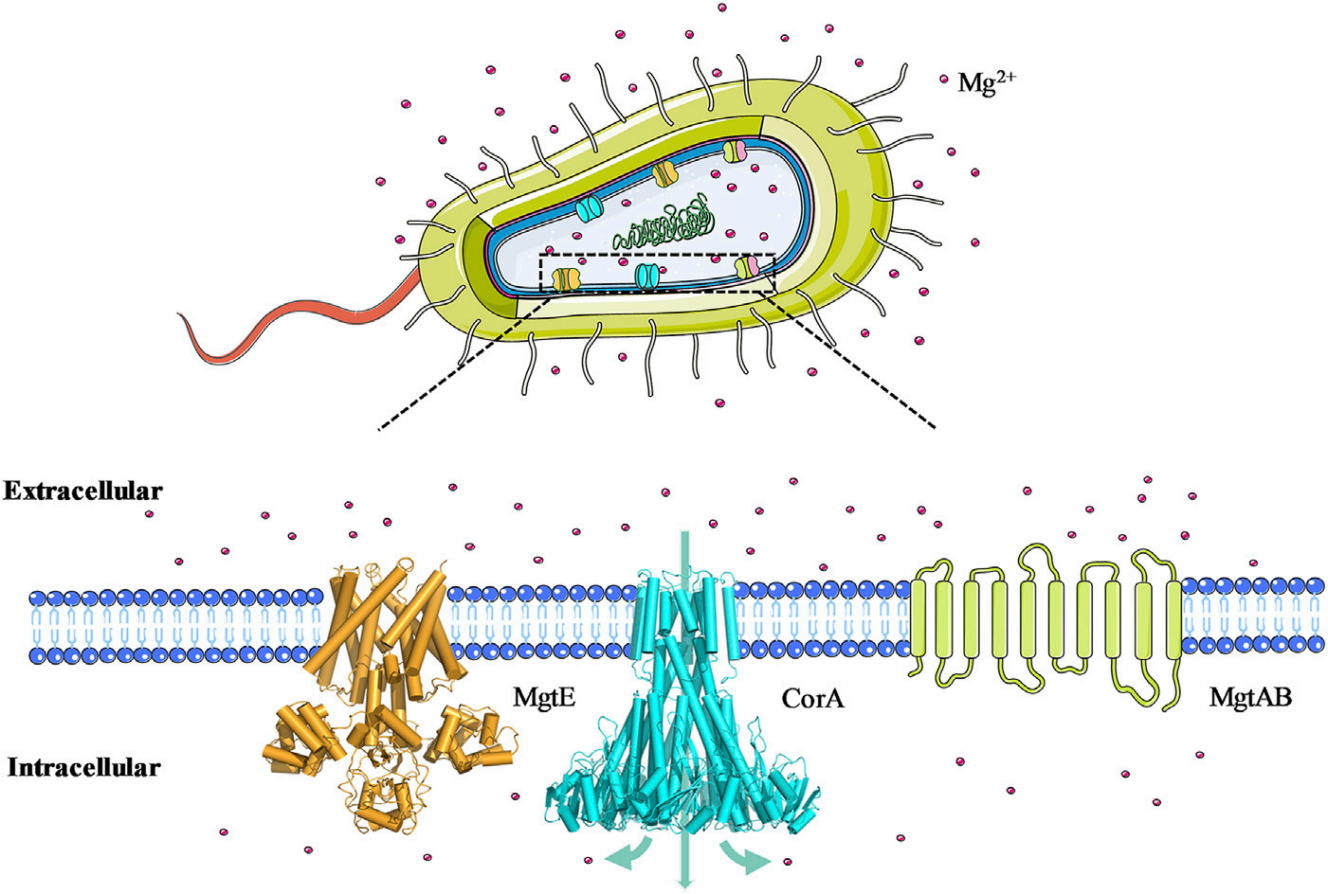
Schematic representation of three prokaryotic Mg^2+^ transporter classes. MgtE is a homodimer in which the cytoplasmic portion of each monomer consists of an N domain, a tandemly repeated cystathionine *β*-Synthase domain. CorA is a homo-pentamer in which each monomer contains a large N-terminal cytoplasmic domain and two transmembrane helices. MgtA is a monomeric P-type ATPase, and its three-dimensional structure has not yet been resolved.

Magnesium ion (Mg^2+^) is one of the most abundant cellular divalent cations and plays a crucial role in numerous biological processes and enzymatic reactions in both prokaryotes and eukaryotes (Romani, 2011). Therefore, regulating and maintaining magnesium homeostasis is a vital prerequisite for the complicated functions of life. All organisms have evolved diverse mechanisms to control the intracellular Mg^2+^ concentration, thereinto, Mg^2+^ transporters and channels are the fundamental influx/efflux machineries *in vivo* (Goytain and Quamme, 2005). Until now, the most extensively investigated Mg^2+^ transporters are from prokaryotic sources and are grouped into three distinct classes (Figure 1): MgtA/MgtB-type transporters belong to P-type ATPases that mediate Mg^2+^ influx down its electrochemical gradient (Maguire, 1992); MgtE-type transporters exist as a homodimer with a 4-5 transmembrane (TM) domains and an N-terminal cytoplasmic domain per monomer, which function as Mg^2+^ importer but lack NTP binding motifs to perform energy-dependent conformational switching (Takeda et al., 2014). CorA-type channels, similar to MgtE, are ion channels composed of a small TM domain and a large cytosolic Mg^2+^ sensor domain (Eshaghi et al., 2006), whereas CorA is a funnel-shaped homopentamer and CorA family members are found to conduct influx of Mg^2+^, Ni^2+^, and Co^2+^ (Xia et al., 2011). Due to their structural diversity, magnesium transporters from different families utilize distinct biochemical mechanisms to perform Mg^2+^-influx/efflux functions. CorA is the most wide-spread and primary Mg^2+^ uptake system in prokaryotes, and in eukaryotes it also maintains the balance of Mg^2+^ uptake in mitochondria, the absence and disruption of which is associated with the emergence of diseases. Therefore, the study of the CorA magnesium transport process and its gating mechanism is essential to understand the occurrence of magnesium disorder-related diseases (Kuramoto et al., 2011).

CorA protein represents a unique cation channel family including yeast magnesium channel Mrs2/Alr1 and *salmonella typhimurium* Zn^2+^ channel ZntB (Khan et al., 2013). They are structurally characterized by 2-TM helices separated by a conserved loop containing GXN motif (Gati et al., 2017). This structural signature and the following MPEL motif are predicted to be essential for the divalent cation selectivity filtering (Hu et al., 2009). The oligomerized C-terminal TM1 and TM2 form the hydrophobic transmembrane channel, while the large hydrophilic N-terminal domain act as an allosteric gating module *via* their specific magnesium sensing and binding features (Maguire, 2006). The opening of the CorA-type channel depends on the intracellular magnesium ion concentrations. Crystal structures of CorA from *Thermotoga maritima* (TmCorA) suggested that CorA maintains a highly symmetrical closed state when magnesium ions bind to the cytoplasmic domain (Pfoh et al., 2012). Crystallography has revealed the three-dimensional structure of CorA and preliminarily elucidated the structural basis of its regulation of magnesium ion channels. However, the limitations of crystallography on samples preparation make it impossible to study the state of CorA in solution, and the molecular weight of CorA protein makes it much more challenging to be studied. Thus, molecular dynamics simulations have been applied to mimic the dynamic changes in the process of CorA channels exerting their functions. The dynamic analysis indicated asymmetric open states exist when the magnesium ion concentration decreases and dissociates from the cytoplasmic domain (Rangl et al., 2019). However, experimental confirmation of the gating mechanism of CorA is still lacking. Subsequently, Cryo electron microscopy (Cryo-EM) structures of TmCorA further revealed an unexpected loss of symmetry within the cytoplasmic domain during the transition between closed and open states (Matthies et al., 2016). But the highly flexible properties of CorA channels in the open state also limited the visualization of the molecular details by cryo-EM. In addition, Functional analyses in bacterial and liposome assays have attempted to verify and discover the molecular characteristics and mechanisms of CorA regulation by magnesium ions (Payandeh et al., 2008; Lerche et al., 2017). In summary, structural and biochemical data have indicated that the subunit interfacial magnesium binding sites play pivotal roles in regulating and driving the states transition of CorA channels (Pfoh et al., 2012). Nevertheless, detailed studies on how CorA channels are regulated by the sensing properties of magnesium ion concentration and the transition of dynamic regulatory processes are still insufficient.

To further investigate the gating behavior of CorA, we selected a putative cation channel Bpss1228 from *B. pseudomallei* as the research object (Techawiwattanaboon and Chareonsudjai, 2016). Sequence alignment and molecular modeling indicate that the Bpss1228 is a distinctive member in CorA family. Bpss1228 was heterologously expressed and purified in *Escherichia coli,* electrophysiological experiments were carried out *in vitro*, which confirmed that Bpss1228 is a strict cation-selective channel and prefer to recognize magnesium ion. Meanwhile, we observed a specific gating behavior of Bpss1228 embedded in the planar lipid bilayer. These results confirm that CorA is open and rapidly uptake magnesium ions when under low magnesium ion conditions, while the high magnesium ion concentration leads to the closure of CorA channels. Moreover, in the electrophysiological experiment, it was found that magnesium ions mediated the formation of multiple states of Bpss1228 proteins before they eventually converged to the closed state, which further confirmed the synergistic regulation of CorA by magnesium ions.

## Materials and methods

### General materials

Chemicals were obtained from Sigma-Aldrich unless stated otherwise.

### Sequence alignment and homology modelling

The analysis of the amino-acid sequence homology and the multiple alignments of protein sequences were achieved using BLASTP database, Bioedit program. Furthermore, the alignment depiction was realized using ESPript 3.0 (https://espript.ibcp.fr/ESPript/cgi-bin/ESPript.cgi). Accordingly, the automated comparative protein structure homology modeling server SWISS-MODEL (http://www.swissmodel.expasy.org), was employed to produce a 3D model of Bpss1228 based on the Cryo-EM structure of CmaX from *Pseudomonas aeruginosa* (PDB ID: 7NH9, structure at 3.03 Å resolution) that was extracted from the Protein Data Bank (http://www.rcsb.org). The molecular graphics system PyMOL v0.99 (http://www.pymol.org) was used to analyze the 3D model and generate the figure.

### Gene cloning and protein purification

The gene of *Bpss1228* was amplified from the *B. pseudomallei* genomic DNA by polymerase chain reaction (PCR) using gene-specific primers. The genes were inserted into the plasmid (pET22b-6His) by using ClonExpress II One Step Cloning Kit (Vazyme). *E. coli* BL21 (DE3) cells, containing pET22b-Bpss1228-6His, were cultured in Luria–Bertani (LB) medium in presence of 50 μg/ml ampicillin at 37°C. When the OD600 reached 0.8–1.0, protein expression was induced with 0.4 mM isopropyl-β-D-thiogalactoside (IPTG) for 16 h at 16°C. Bacteria were collected by centrifugation at 4,000 ×g for 15 min and resuspended in lysis buffer A (25 mM Tris, 150 mM NaCl, pH 7.8). The cells were disrupted and lysed by high-pressure with 1 mM phenylmethylsulfonyl fluoride (PMSF). After that, the supernatant was obtained by centrifugation at 8,000 ×g for 30 min and added 1% (wt/vol) n-Dodecyl-β-D-maltopyranoside (DDM) to extraction for 2 h. The supernatant was obtained by centrifugation at 18,000 ×g for 30 min and then co-incubated with Ni-NTA resin (Qiagen) for 1 h. The mixture was washed with buffer B (25 mM Tris, 150 mM NaCl, 30 mM imidazole, 0.02% DDM, pH 7.8) and the target protein was eluted with lysis buffer C (25 mM Tris, 150 mM NaCl, 300 mM imidazole, 0.06% glyco-diosgenin (GDN), pH 7.8, 1 mM ethylenediaminetetraacetic acid (EDTA). The protein was further purified with size-exclusion chromatography Superose 6 Increase 10/300 GL (GE Healthcare), which was pre-equilibrated with solution buffer D (25 mM Tris, 150 mM NaCl, 0.06% GDN, pH 7.8). Peak fractions were determined by SDS-PAGE analysis.

### Planar lipid bilayer analysis

The currents of purified Bpss1228 were recorded by on a HEKA patch clamp amplifier (HEKA USB10) with a sampling frequency of 10 kHz at 25°C. Planar lipid bilayers were prepared using *E. coli* Polar Lipid Extract (AVANTI) dissolved in decan. All solution were buffered by 10 mM HEPES (pH 7.5). Bpss1228 proteins were added in cis-chamber to form a single channel. Titration experiments were used to examine the ability of different ions to pass through the CorA channel. After the protein was added to asymmetric solutions (30/300 mM NaCl, trans/cis), the protein was embedded in the phospholipid membrane to form a channel. The membrane potential was then changed to the equilibrium potential of sodium ions. Next, a chloride solution containing 20 mM (final concentration) analyte ions was added to the trans-chamber. If the addition of one type of ion causes a step-like current, it indicates that the channel is permeable to that type of ion. In titration experiments, the currents induced by the addition of divalent ions were normalized to compare their ability to penetrate CorA channels. The signal was filtered with a low-pass Bessel filter at 5 kHz. Clampfit and Origin software were used to analyze the data. All representative current traces were filtered with 800 Hz.

## Result and discussion

### Sequence alignment and molecular modeling reveal that Bpss1228 is a CorA-type channel

**FIGURE 2:**
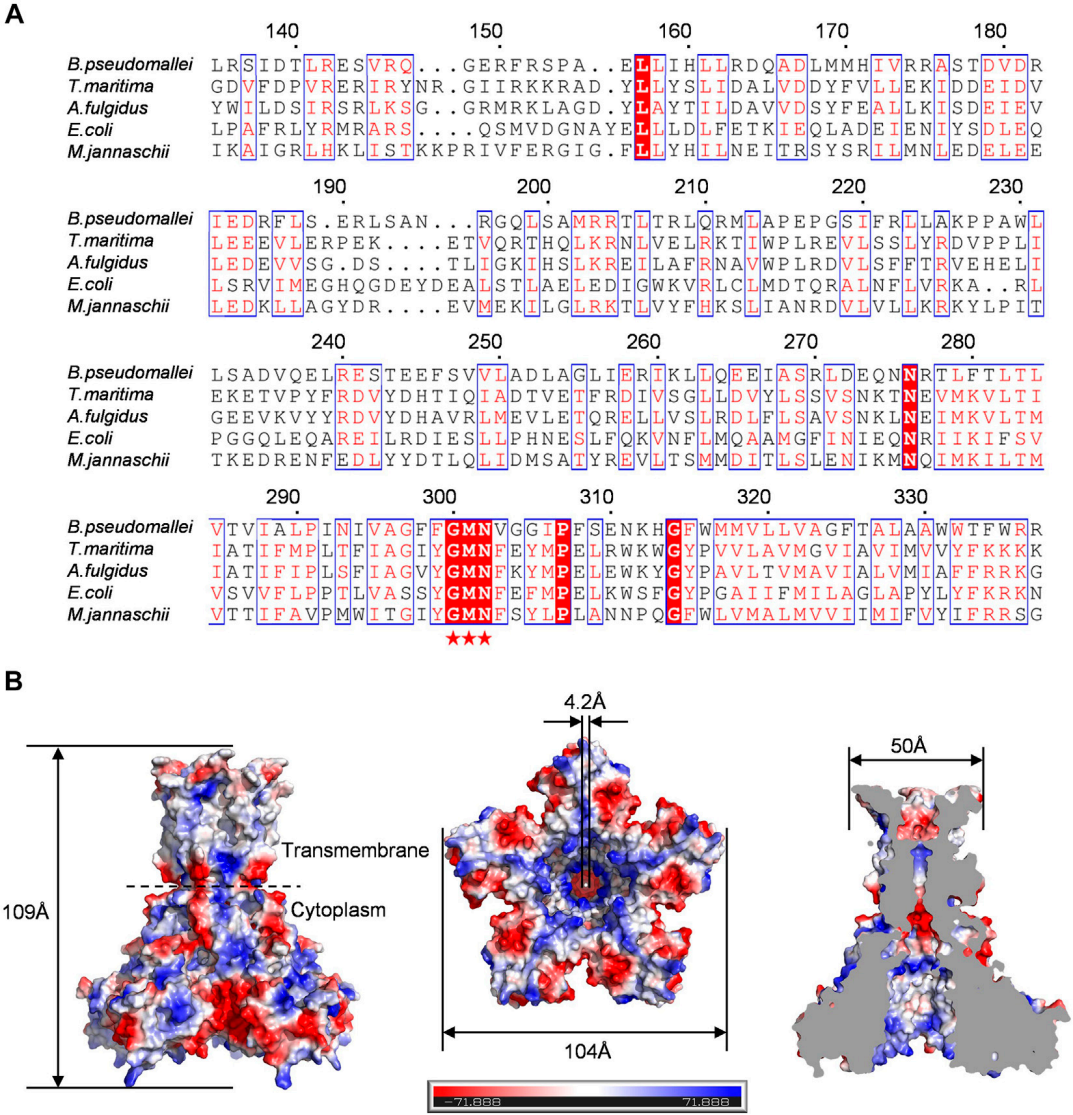
Sequence alignment and molecular modeling confirmed that Bpss1228 is a typical CorA channel. **(A)** Multi-sequence alignment of Bpss1228 with its four homologs. Bpss1228 contains the typical CorA family GMN motif with all homologs, and the GMN motif is marked with a pentagram below. **(B)** The 3D structure of Bpss1228 was created by SWISS-MODEL (https://swissmodel.expasy.org/interactive) through homology modeling (PDB:7NH9 as template). The electrostatic potential surface of the Bpss1228 prediction model, the height of Bpss1228 is about 109 Å, the bottom of the cytoplasmic end is about 104 Å wide, and the extracellular end is about 50 Å wide, forming a channel with an internal diameter of about 4.2 Å at its narrowest point.

To investigate the properties of Bpss1228, we performed a sequence alignment with *T. maritima*. TmCorA (Eshaghi et al., 2006), *E. coli*. EcCorA (Lerche et al., 2017), *Methanocaldococcus jannaschii*. MjCorA (Guskov et al., 2012), and *Archaeoglobus fulgidus*. AfCorA (Payandeh and Pai, 2006) (Figure 2A). According to sequence comparisons, the C-terminal region (279–340) corresponds to a transmembrane domain. It possesses characteristic Glycine-methionine-aspartate (G300, M301, N302) selectivity filter (Palombo et al., 2013)**.** This motif is located at the position between the extracellular loop of the two transmembrane helices of Bpss1228. This signature is one of the most conserved motifs of the CorA family and is related to the ion selectivity. Sequence comparisons indicate that the N-terminal (1–279) primary structure is divergent, but we also note the presence of negatively charged residues in the cytoplasmic regions (D167, D179, D181, E183) of all CorA homologs. It is likely that these residues are conserved due to the specific nature of CorA binding to divalent cations. Furthermore, multiple but slightly different magnesium binding sites were identified in both TmCorA and MjCorA, which are the molecular basis for intracellular Mg^2+^ sensing and allosteric gating regulation. This is consistent with the widespread of negatively charged residues in Bpss1228, suggesting that it is also regulated by magnesium ions.

Structural model of Bpss1228 was generated by the SWISS-MODEL server using *P. aeruginosa* CmaX as template (Stetsenko et al., 2021). This template was selected on the basis of the highest percentage of identity and sequence coverage of Bpss1228, and the best results on the geometry given by the CCP4 analysis. Consequently, the two sequences shared an identity of 26.44% with a coverage of 0.97. The Ramachandran plot analysis showed that 90.55% of the residues are in the favored regions and 94.82% are in the allowed regions. C-alpha atom root mean square deviation (RMSD) between the superposed Bpss1228 homology model and the 7NH9 template was of 0.21 Å, suggesting the dependability of the predicted structure in further analysis. The complete assembled Bpss1228 model has a total length of 109 Å along the five-fold axis, and a maximum width of 104 Å perpendicular to the five-fold axis (Figure 2B). The Bpss1228 homo-pentamer forms a funnel-shaped ion-permeable channel, at its widest, 18 Å in diameter, and at its narrowest, 4.2 Å. This model represents the close-state structure of the CorA channel. The overall structure of Bpss1228 showed a homopentameric arrangement with a large intracellular hydrophilic domain and a relatively small C-terminal transmembrane domain. As shown in the electrostatic surface potential map, there are a number of negatively charged amino acid residues (E149, E156, D181, D185, and D235) located at the monomer-monomer interfaces grooves, which are proposed to bind the Mg^2+^. Similar sites in the crystal structures of TmCorA, MjCorA, EcCorA and the Mg^2+^ transporter MgtE have been found, which have been postulated as the regulatory binding sites (Ishitani et al., 2008). In addition, the ions selectivity filter GMN motif of Bpss1228 is positioned at the extracellular loop between TM1 and TM2, which is also consistent with the identified CorA family channel profile. Based on these analyses, we revealed that Bpss1228 is a CorA-type channel and its physiological function may be regulated by Mg^2+^, and we can further investigate the gating behavior of this channel.

### The Bpss1228 protein forms a stable ion channel in planar lipid bilayers

**FIGURE 3:**
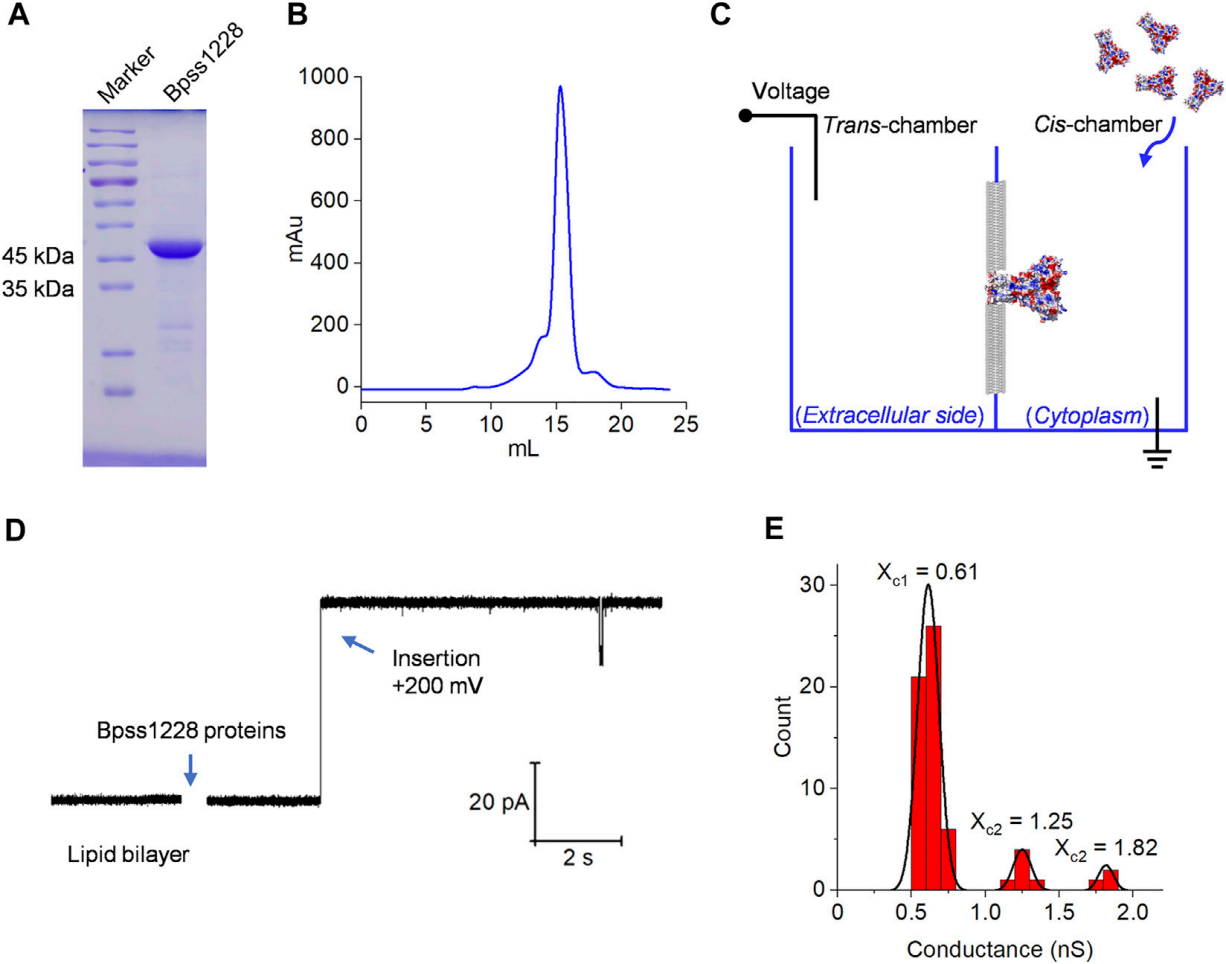
The Bpss1228 forms an ion channel in planar lipid bilayers. **(A)** 12% SDS-PAGE of Ni-resin purified Bpss1228, visualized with Coomassie brilliant blue, the MW of Bpss1228 is 38.5 kDa. **(B)** Size-exclusion chromatography analysis of the Bpss1228. The proteins were analyzed in the Superose 6 10/300 GL column (GE Healthcare). **(C)** The schematic illustration of lipid bilayer recording. **(D)** The CorA insertions in planar lipid bilayers at 200 mV. **(E)** Histogram of the conductance values measured for the CorA (*n* = 62). The line represents a Gaussian fit to the binned data (bin width = nS). The three peaks represent three different conductivity distributions. This experiment was conducted in 30/300 mM (trans/cis) NaCl, both buffered with 10 mM HEPES, pH 7.5.

The planar lipid bila**y**er experiment is a useful technique for studying ion selectivity and gating behavior of channel-forming proteins such as ZAR1 protein (Bi et al., 2021), phi29 connector (Geng et al., 2011), PANX1 (Mou et al., 2020) and TACAN channel (Ke et al., 2021), etc. The Bpss1228 full-length protein was over-expressed in *E. coli* BL21 (DE3) and purified by Ni-chelating chromatography. Based on the results of sodium dodecyl sulfate-polyacrylamide gel electrophoresis (SDS-PAGE) and gel filtration (Figures 3A,B). The protein eluted as a single predominant peak at 15.1 ml on a Superose 6 Increase 10/300 GL analytical gel filtration column, corresponding to an apparent molecular weight of 200 kDa, suggesting a homopentameric state of Bpss1228. The schematic diagram of the planar lipid bilayer experiment is shown in Figure 3C. Purified Bpss1228 proteins were added in cis-chamber after forming stable planar lipid bilayers. When incorporated into lipid membranes, Bpss1228 proteins exhibit channel activity with stable current. Depending on the directionality of the channel, the cis-chamber is considered the cytoplasmic side, while the trans-chamber is considered the extracellular side. A step-wise current increase was observed when the Bpss1228 protein was inserted into membranes at 200 mV, implying successful construction of the channel (Figure 3D). We calculated the conductance of Bpss1228 channels by fitting to Gaussian functions (Figure 3E) (*n* = 62), and the fitted curve shows three distinct peaks at 0.61 nS (53/62), 1.25 nS (6/62) and 1.82 nS (3/62), which is obviously due to the different number of Bpss1228 channels inserted in membranes, as these values increase in approximately equal proportions. With this statistical method, we consider the conductance of a single CorA-channel is 0.61 nS in an asymmetric 30/300 mM (trans/cis) NaCl buffer condition.

### The Bpss1228 protein possesses cation-selective channel activity in planar lipid-bilayers

**FIGURE 4:**
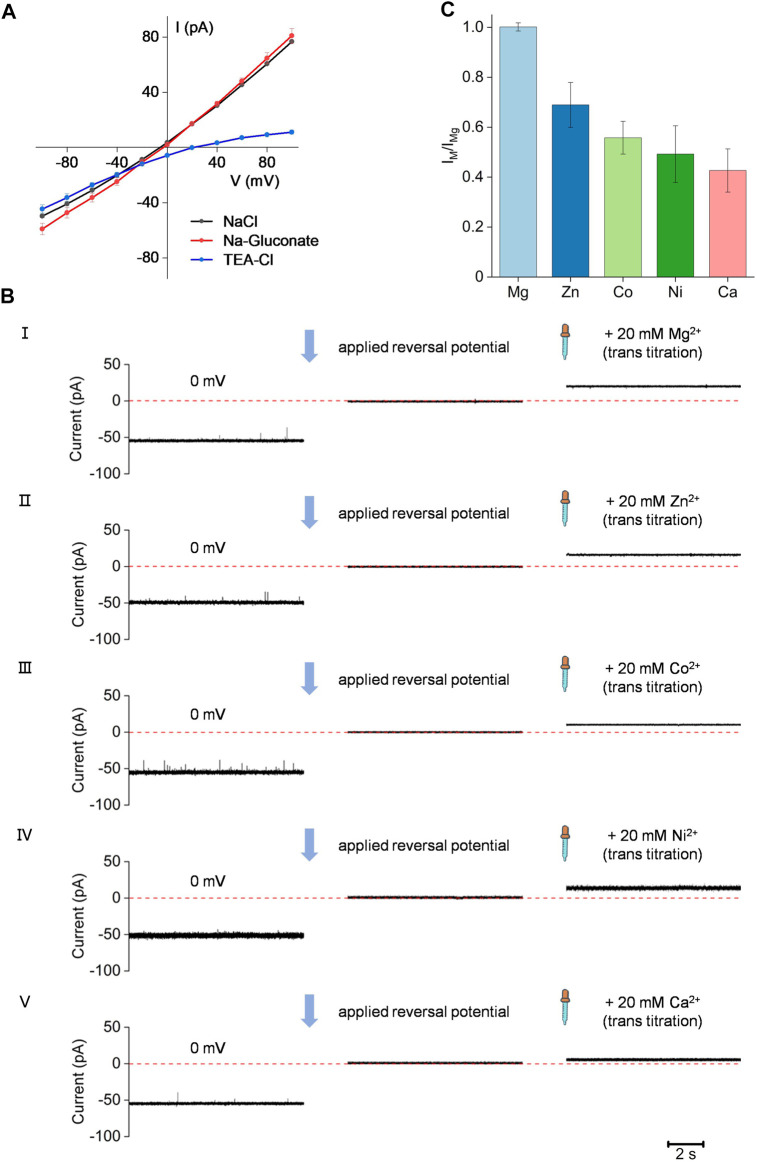
The Bpss1228 channel is permeable to cation. **(A)** The current-voltage relationship of the CorA demonstration cation selectivity. Cis-chamber solutions are 150 mM NaCl, and the different color of dots represent different buffers in trans-chamber, black dots (150 mM NaCl), red dots (150 mM Na-Gluconate) and blue dots (150 mM TEA-Cl), all buffered with 10 mM HEPES, pH 7.5 (Mean ± SE, *n* = 3). **(B)** The represent current traces applied at 0 mV or reversal potential, and added in 20 mM divalent cations. From I to V represent Mg^2+^, Zn^2+^, Co^2+^, Ni^2+^, Ca^2+^, respectively. The cis-chamber contained 300 mM NaCl, and the trans-chamber was loaded with 30 mM NaCl, all buffered with 10 mM HEPES, pH 7.5. **(C)** Bar chart showing different current change after adding the divalent cations described in **(B)** (Mean ± SE, *n* = 3).

To better study the channel activity and ion selectivity of the Bpss1228 protein, we conducted single-channel measurements in symmetrical or asymmetrical buffer conditions (Figure 4A). In the symmetrical buffer conditions of 150 mM NaCl in both trans and cis chamber, applying a voltage of −100 to 100 mV, it can be seen that the I-V curve is linear (black dots), confirming that the Bpss1228 protein formed an ion-conducting channel in the membranes. To further examine the permeability property of the Bpss1228 channel, the 150 mM NaCl in the trans-chamber was replaced with 150 mM Na-Gluconate or tetraethylammonium chloride (TEA-Cl). We also observed a linear I-V curve in 150 mM Na-Gluconate buffer conditions, suggesting that Na^+^ could permeate through the Bpss1228 channel, as gluconate is an impermeable anion, whose dimension is much larger than 4 Å at the narrowest constriction of the CorA channel (Hattori et al., 2007). When we replaced the 150 mM NaCl buffer in the trans-chamber with the 150 mM TEA-Cl buffer, however, a distinct I-V curve was observed. When a negative voltage was applied, the current-voltage relationship is linear, while when a positive voltage was applied, the current change is non-linear and small. TEA is a large cation with a radius of 3.85 Å and is not permeable to the CorA channel (Neale et al., 2015). This result suggest that the CorA channel is hardly permeable to chloride anions as very little positive current was observed under positive voltages applied. In summary, all these results demonstrate that the CorA channel is permeable to cation.

Ion specificity is a basic property of ion channels. To evaluate ion specificity of Bpss1228 channel, we calculated the selectivity of Bpss1228 channel for various divalent cations by titration assays (Figure 4B) (Sun et al., 2018). In the titration assays, the Bpss1228 channel had a current around -50 pA at 0 mV in an asymmetric 30/300 mM (trans/cis) NaCl buffer condition. The current was regulated at 0 pA by applying a reversal potential, and then tested different bivalent cations individually for their ability to permeate across Bpss1228 channel by adding 20 mM chloride to the trans-chamber. The result shows that Bpss1228 channel could translocate Mg^2+^, Zn^2+^, Co^2+^, Ni^2+^, and Ca^2+^. The order of the relative cation permeability of CorA channel is as follows: current change I_Mg_ > I_Zn_ > I_Co_ > I_Ni_ > I_Ca_ (Figure 4C). Taken together, the results of these electrophysiological experiments demonstrate that Bpss1228 is a magnesium channel.

### Step-wise gating of the CorA channel triggered by Mg^2+^

**FIGURE 5:**
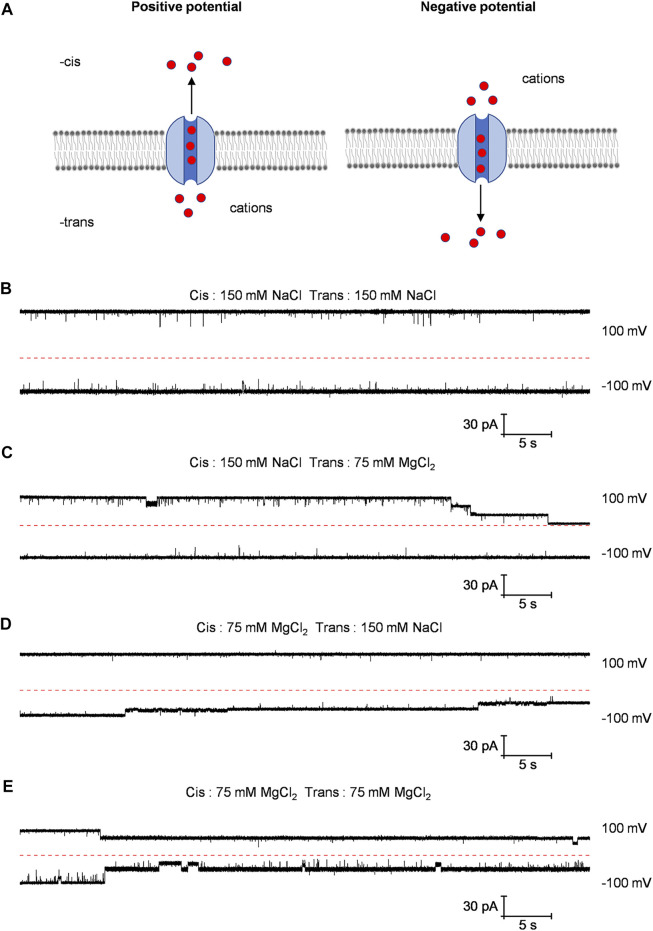
Different current traces of Bpss1228 channel under varied buffer conditions. **(A)** Schematic diagram of cations passing through the CorA channel when different voltages are applied. **(B–E)** The represent current traces at 100 mV and −100 mV in different buffer conditions as indicated. All buffered 10 mM HEPES, pH 7.5.

Numerous studies have shown that the CorA protein is gated by magnesium ions (Dalmas et al., 2010; Dalmas et al., 2014). In order to study the gating of the CorA channel embedded in planar lipid bilayer, we recorded the currents of the Bpss1228 channel under four different buffer conditions at 100 mV and −100 mV (Figures 5B–E). Not surprisingly, under the symmetrical buffer conditions of the 150 mM NaCl in both trans and cis chamber, the current traces not have distinct characteristics as sodium ions only pass through the channel rather than interact with it (Figure 5B). When the 150 mM NaCl in the trans-chamber was replaced with the 75 mM MgCl_2_, the step-wise gating was observed at 100 mV, but not observed at −100 mV (Figure 5C). From the schematic diagram (Figure 5A), it can be seen that when a positive potential is applied, cation flow from the trans-chamber to the cis-chamber through the Bpss1228 channel, and vice versa. Therefore, the presentative current traces suggest that magnesium ions through the channel may be responsible for the gating. To demonstrate this conjecture, we replaced the 150 mM NaCl in the cis-chamber with the 75 mM MgCl_2_ (Figure 5D) and, unsurprisingly, the current was gated at −100 mV, not at 100 mV. Similarly, when the 150 mM NaCl in the both cis-chamber and trans-chamber were replaced by the 75 mM MgCl_2_, the channel gating occurred at either 100 or −100 mV (Figure 5E). In these experiments, the applied voltage was only used to modulate the transport direction of Mg^2+^ though the channel, and the gating was affected by Mg^2+^ rather than the sequence of applied voltages.

**FIGURE 6:**
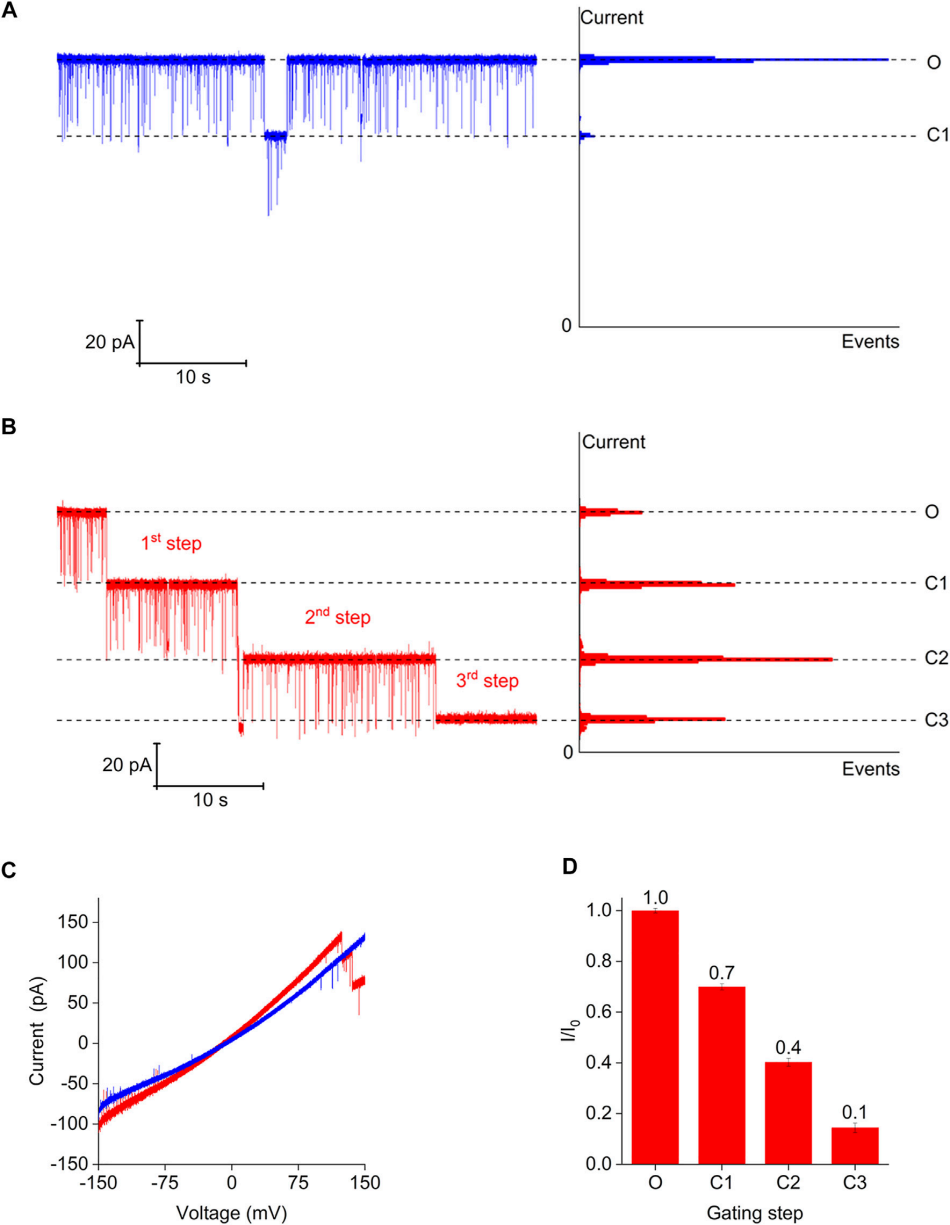
Step-wise gating of Bpss1228 channel triggered by Mg^2+^. **(A)** Representative current traces of the CorA channel at 150 mV in symmetry 150 mM NaCl solution, all chamber buffered with 10 mM HEPES, pH 7.5. All-points amplitude histogram generated from the continuous single-channel recording. **(B)** Representative step-wise current traces of the CorA channel in symmetry 150 mM NaCl solution, but the trans-chamber added in 10 mM MgCl_2_, and all chamber buffered with 10 mM HEPES, pH 7.5. All-points amplitude histogram generated from the continuous single-channel recording of. “O,” “C1,” “C2,”and “C3” indicate open state and step-wise state of the CorA channel. **(C)** High ramping potential in different buffer conditions, and blue line represents **(A)**, red line represents **(B)**. **(D)** Histogram show the number and size of gating step. (Mean ± SE, *n* = 3).

To further demonstrate the channel gating induced by magnesium ions and describe the channel gating behavior, we recorded representative current traces of the Bpss1228 channel under two different buffer conditions at 150 mV (Figures 6A,B). The Bpss1228 protein embedded in the planar lipid bilayer was stable and displayed a uniform conductance at 150 mV under the symmetrical buffer conditions of the 150 mM NaCl, even if the Bpss1228 channel has a small step “C1” of about 30%, it will quickly recovery. These results indicates that the CorA protein is relatively stable under this condition, without significant conformational changes, and in the absence of magnesium ions, the channel does not interact with ions (Figure 6A). But when the trans-chamber contains 10 mM magnesium ions under the symmetrical buffer conditions of the 150 mM NaCl in the both trans and cis chamber, the Bpss1228 channel exhibited typical step-wise gating behavior (Figure 6B). The ramping potential in the above buffer conditions shows that in the presence of magnesium ions, magnesium ions could interact with the channel at high voltage, resulting in a conformational change of the channel, which shows a significant gating phenomenon in the electrophysiological experiment (Figure 6C). In the presence of magnesium ions, three-step was observed from the open state to the close state, each reduction step size being approximately 30% of the Bpss1228 channel. In previous studies two asymmetric open conformation CorA structures revealed the dynamic gating mechanism of CorA in Mg^2+^-free solutions (Matthies et al., 2016). In electrophysiological experiments we found that CorA has a strong ion permeability in the open state, which is consistent with the observations in the open state conformation structures. In addition, three-steps gating also revealed the existence of three open states of CorA on planar lipid bilayer, which is inconsistent with only two open conformations observed in the Cryo-EM structure, and we speculate that this is due to the single-particle classification process of the Cryo-EM structure not being able to contain all open conformations. Interestingly, after the three-step reduction, there was still a residual current opening, equal to ∼10% of the Bpss1228 channel (Figure 6D). This demonstrates that even in the closed state, the CorA channel retains a certain pathway for ion conduction, which is in accordance with the permeation pathways measured in the crystal structures of various CorA-Mg^2+^ complexes (Lunin et al., 2006; Tan et al., 2009). In conclusion, these results confirmed that gating of the CorA channel regulated by magnesium ions. And the step-wise gating behavior revealed the conformation change of CorA with presence and absence magnesium ions.

## Conclusion

CorA family channels play an indispensable role in most biological magnesium homeostasis. In this study, we characterized the Bpss1228 protein as a typical CorA-type channel by sequence comparison and homology modeling. Subsequently, we purified the Bpss1228 protein and further reconstituted it into a planar lipid bilayer *in vitro*. We confirmed that the Bpss1228 has channel activity and uniform conductance, based on these results, we confirmed the Bpss1228 protein is cation-selective channel. Although the current study identifies CorA as a cation-selective channel and little has been reported about its permeability to anions, it will be very interesting to study various other anions. Furthermore, the Bpss1228 channel is permeable to a variety of divalent cations tested including Mg^2+^, Zn^2+^, Co^2+^, Ni^2+^, and Ca^2+^. Finally, we found that Bpss1228 is not only selectively permeable to magnesium ions in electrophysiological experiments, but also exhibits a specific gating behavior when encountering magnesium ions. Generally, we demonstrated a stepwise gating behavior of Bpss1228 on planar lipid bilayers and further confirmed the gating mechanism of CorA family proteins regulated by magnesium ions. Our results indicate that the gating behavior induced by magnesium ions is sequential, suggesting that magnesium ions act as ligands to progressively stabilize adjacent subunits of CorA.

## Data Availability

The original contributions presented in the study are included in the article/supplementary material, further inquiries can be directed to the corresponding authors.
